# Binary stress induces an increase in indole alkaloid biosynthesis in *Catharanthus roseus*

**DOI:** 10.3389/fpls.2015.00582

**Published:** 2015-07-28

**Authors:** Wei Zhu, Bingxian Yang, Setsuko Komatsu, Xiaoping Lu, Ximin Li, Jingkui Tian

**Affiliations:** ^1^Institute of Biomedical Engineering, College of Biomedical Engineering & Instrument Science, Zhejiang UniversityHangzhou, China; ^2^National Institute of Crop Science, National Agriculture and Food Research OrganizationTsukuba, Japan; ^3^Ministry of Education Key Laboratory for Biomedical Engineering, Zhejiang UniversityHangzhou, China

**Keywords:** biosynthesis pathway, *Catharanthus roseus*, dark incubation, indole alkaloids, gel-free proteomics, UV-B irradiation

## Abstract

*Catharanthus roseus* is an important medicinal plant, which produces a variety of indole alkaloids of significant pharmaceutical relevance. In the present study, we aimed to investigate the potential stress-induced increase of indole alkaloid biosynthesis in *C. roseus* using proteomic technique. The contents of the detectable alkaloids ajmalicine, vindoline, catharanthine, and strictosidine in *C. roseus* were significantly increased under binary stress. Proteomic analysis revealed that the abundance of proteins related to tricarboxylic acid cycle and cell wall was largely increased; while, that of proteins related to tetrapyrrole synthesis and photosynthesis was decreased. Of note, 10-hydroxygeraniol oxidoreductase, which is involved in the biosynthesis of indole alkaloid was two-fold more abundant in treated group compared to the control. In addition, mRNA expression levels of genes involved in the indole alkaloid biosynthetic pathway indicated an up-regulation in their transcription in *C. roseus* under UV-B irradiation. These results suggest that binary stress might negatively affect the process of photosynthesis in *C. roseus*. In addition, the induction of alkaloid biosynthesis appears to be responsive to binary stress.

## Introduction

*Catharanthus roseus* (*C. roseus*) produces a wide range of indole alkaloids, many of which are pharmaceutically important compounds (Ganapathi and Kargi, 1990). Vinblastine and vincristine are frequently used as cancer chemotherapeutics (Gidding et al., 1999). Aajmalicine was shown to have anti-hypertensive and anti-arrhythmias activities (Almagro et al., 2011). Vindoline and catharanthine displayed anti-bacterial activities, anti-diabetic properties, and diuretic actions (Chen et al., 2013). Given these biological activities, the indole alkaloids have been comprehensively investigated in the last two decades (Irie et al., 1987; O'Connor and Maresh, 2006), with *C. roseus* as an important and attractive resource for research of anticancer drugs.

Since most of the active components accumulate as secondary metabolites, these compounds are often present in trace quantities in plants, which hinders industrial-scale purification. The insufficiency of indole alkaloids from the available natural sources has led to the development of a number of alternative methods for their synthesis and production, including chemical synthesis (total synthesis or semi-synthesis), metabolic engineering, plant cell, and tissue culture (Hughes and Shanks, 2002). However, there are some major flaws in all of the current alternative methods.

Although chemical synthesis of alkaloids from *C. roseus* had been reported, it was not applicable for industrial-scale production due to its low productivity and high cost. In recent decades, various intermediates of indole alkaloids were identified (St-Pierre et al., 1999), and a series of key enzymes involved in the biosynthesis of indole alkaloids were cloned and characterized (Han et al., 2007). In addition, the metabolic network of indole alkaloids has been vigorously studied (Richer et al., 2006), making it possible to improve the content of alkaloids in *C. roseus* by metabolic engineering (van der Heijden et al., 2004). For example, overexpression of *ORCA3* and *G10H* genes in *C. roseus* largely increased the accumulation of vindoline, catharanthine and ajmalicine (Pan et al., 2012). Using transgenic plants for the production of alkaloids has also been explored, however, genetic instability is a major hindrance in its application for commercial production (Koprek et al., 2001). Although large-scale *C. roseus* cell culture can also be used to produce the alkaloids, the levels of production were still insufficient for commercial production (Mujib et al., 2012). Moreover, due to the absence of certain enzymes, several important alkaloids such as the bisindole alkaloids, cannot be produced in cell cultures (Kutchan, 1995).

The rate of biosynthesis of secondary metabolites in plants is usually influenced by abiotic stress (Ramakrishna and Ravishankar, 2011). The content of catharanthine in *C. roseus* plant cell suspension was increased by exposure to a low dose of ultraviolet-B (UV-B) irradiation (Ramani and Chelliah, 2007). When *C. roseus* hairy roots were exposed to UV-B light for 20 min, the concentrations of lochnericine, serpentine, and ajmalicine were increased by 60, 20, and 50%, respectively (Binder et al., 2009). The reported results verified that UV-B was an elicitor of indole alkaloids. Nevertheless, the effect of UV-B irradiation on the content of indole alkaloids in *C. roseus* at the whole plant scale, which is the sole source for commercial production of alkaloids, has not yet bee investigated.

Recently, comparative proteomics has been widely applied in the investigation of biosynthetic mechanisms in plants. For *C. roseus*, the proteomic analysis of suspension cell had been carried out using two-dimensional electrophoresis, two-dimensional fluorescence difference gel electrophoresis, and gel-free approaches (Jacobs et al., 2005; Champagne et al., 2012). The results indicated that gel-based proteomic technique had the limitation to detect low-abundant proteins. Gel-free quantitative proteomics has since been rapidly developed, and is more useful for the identification of a large number of proteins (Neilson et al., 2011). This approach provides significantly more detailed proteome information on low-abundant proteins and might be more suitable for the investigation of proteins involved in secondary metabolism in plants.

In this study, the effects of UV-B irradiation and dark incubation on alkaloid contents, mRNA expression, and protein abundance were analyzed to investigate the influence of binary stress on the regulation of alkaloid biosynthesis. The content of the detectable alkaloids, ajmalicine, vindoline, catharanthine, and strictosidine as well as various physiological parameters were analyzed in the leaves of *C. roseus* after UV-B irradiation and dark incubation. Subsequently, the physiological parameters of *C. roseus* leaves were analyzed. To better understand the UV responsive mechanism in *C. roseus*, gel-free/label-free proteomics was performed. Moreover, the expression patterns of key genes involved in alkaloid biosynthesis were analyzed to gain further insights into the molecular mechanism of increased indole alkaloid biosynthesis in *C. roseus.*

## Material and methods

### Plant materials and treatments

*Catharanthus roseus* was provided from College of Pharmacy, Zhejiang University (Hangzhou, China). The plants were cultivated in a greenhouse for 45 days after sowing with temperature maintained at 25–28°C and 80% relative humidity. For UV-B irradiation, the light intensity of UV-B (1345.00 μW·cm^−2^) was measured from 275 to 320 nm with an UV radiometer (Beijing Normal University photoelectric instrument factory, Beijing, China). For the analysis of alkaloid content, the plants were exposed to UV-B for 1, 2, and 3 h, and then cultured for 72 h in darkness. The leaves were dried at 50°C in a drying oven and ground into a coarse powder for 1 min using a high-speed mixer. The coarse powder was screened into fine powder using a mesh with a 0.43 mm aperture. For the analysis of physiological parameters, the plants were exposed to UV-B for 1 h followed by a 72 h dark incubation prior to harvesting the leaves. For proteomic analysis, the plants were exposed to UV-B for 1 h followed by a 72 h dark incubation. Leaves were then collected, frozen in liquid nitrogen, and stored at −80°C. For quantitative reverse transcription-polymerase chain reaction (qRT-PCR) analysis, the plants were exposed to UV-B for 0, 15, 30, and 60 min without dark incubation. The fresh leaves were frozen in liquid nitrogen and stored at −80°C. The control groups of all experiments were incubated in the dark for 72 h without prior UV-B irradiation. For all experiments, three independent experiments were performed as biological replicates (Supplementary Figure 1).

### Quantitative analysis of alkaloids

Samples (1 g dry weight) were extracted in 100 mL of methanol using an ultrasonic extraction method (Tanabe et al., 1999) for three times (for 1 h each). The methanol extracts were dried in a rotary evaporator at 50°C. The residue was dissolved in 5 mL of methanol, and then filtered through a 0.45-μm filter (Millipore, Bullerica, MA, USA) for high performance liquid chromatography (HPLC) analysis. Several standard compounds, which consisted of strictosidine, vindoline, catharanthine, and ajmalicine were provided by Zhejiang Institute for Food and Drug Control (Hangzhou, China). For quantification, a calibration curve was constructed using the standard solutions diluted in methanol at six different concentrations: strictosidine (0.125, 0.250, 0.375, 0.500, 0.625, and 0.750 mg·mL^−1^), vindoline (0.181, 0.362, 0.543, 0.724, 0.905, and 1.086 mg·mL^−1^), catharanthine (0.098, 0.196, 0.294, 0.392, 0.490, and 0.588 mg·mL^−1^), and ajmalicine (0.035, 0.070, 0.105, 0.140, 0.175, and 0.209 mg·mL^−1^). For HPLC analysis, 20 μL of the standard solutions and samples were used.

HPLC analysis was performed on a Waters 2695 Alliance HPLC system (Waters, Milford, MA, USA) equipped with a photodiode array detector. The compounds were separated using reversed phase high performance liquid chromatography. A C18 column (250 × 4.6 mm, Waters) was used with a flow rate of 1 mL·min^−1^ at 40°C. Gradient elution was employed for qualitative and quantitative analyses using mobile phase of 0.025 M ammonium acetate solution and acetonitrile: 0–40 min (30–60% acetonitrile) and 41–50 min (60–80% acetonitrile). Spectra were measured at a wavelength of 220 nm, and peaks were determined by comparing the retention time and UV spectra with those of the standards.

### Analysis of the physiological parameters

Photosynthetic pigment content was determined according to the procedures of Lichtenthaler and Wellburn (1983). Malondialdehyde (MDA) content was assayed (Heath and Packer, 1968) with minor modifications. In order to determine the MDA content, a portion (0.3 g fresh weight) of leaves was ground in 3 mL of 10% trichloroacetic acid and centrifuged at 5000 × g for 10 min. Then, 2 mL of 0.6% thiobarbituric acid was added to 2 mL of the obtained supernatant. The mixture was kept in boiling water for 15 min, and the absorbance of the solution was measured at wavelengths of 532, 450, and 600 nm.

The contents of soluble sugar and soluble protein were determined by the anthrone method (Fales, 1951) and the Lowry method (Lowry et al., 1951), respectively. The proline content was measured according to published method (Bates et al., 1973). Briefly, a portion (0.3 g) of leaves was homogenized in 3% aqueous sulfosalicylic acid. Then it was centrifuged at 5000 × g for 10 min. A reaction mixture consisting of 2 mL of supernatant, 2 mL of ninhydrin and 2 mL of glacial acetic acid was boiled at 100°C for 1 h. The resulting reaction mixture was extracted with 5 mL of toluene and the absorbance of the proline-ninhydrin chromophore was measured at 520 nm.

The superoxide dismutase (SOD) activity was assayed using the SOD assay kit-WST (Dojindo Molecular Technologies, Gaithersburg, MD, USA) according to the manufacturer's protocol. Briefly, the formation of a formazan dye upon reduction of the tetrazolium salt WST-1 (2-(4-iodophenyl)-3-(4-nitrophenly)-5-(2, 4-disulfophenyl)-2H-tetrazolium) with superoxide anions was measured. The mixture of the supernatant and enzyme working solution was incubated for 20 min at 37°C and the absorbance was measured at 450 nm.

Peroxidase (POD) activity was assayed using the POD assay kit (Jiancheng Bioengineering Institute, Nanjing, China) according to the manufacturer's protocol. Briefly, a portion (0.3 g) of leaves was homogenized in 5 mL of 0.1 M phosphate buffer (pH 6.8) and then centrifuged at 15,000 × g for 15 min. The supernatant was collected as enzyme extract. A mixture of 0.125 mM phosphate buffer (pH 6.8), 0.05 mM pyrogallol, 0.05 mM hydrogen peroxide, and 1 mL of enzyme extract was incubated for 5 min at 25°C and the absorbance was measured at 420 nm.

The glutathione content was determined using a Reduced Glutathione Detection Kit (Jiancheng Bioengineering Institute, Nanjing, China) according to the manufacturer's protocol. Briefly, a portion (0.3 g) of leaves was homogenized in 3 mL of 10% trichloroacetic acid containing 5 mM EDTA and was then centrifuged at 12,000 × g for 20 min. The supernatant was collected. Then, 1 mL of the obtained supernatant were added to the tubes containing 1 mL of 0.1 M phosphate buffer (pH 7.7), 20 μL of 40 mM 5, 5-dithiobis (2-nitrobenzoic acid), 20 μL of 10 mM NADPH. The reaction was started by the addition of 20 μL glutathione reductase. After mixing, the absorbance was monitored for 5 min at 412 nm.

### Protein extraction

Samples (0.5 g fresh weight) were ground to powder in liquid nitrogen and then transferred into a polypropylene tube containing an acetone solution of 10% trichloroacetic acid and 0.07% 2-mercaptoethanol. The resulting mixture was vortexed and then sonicated for 5 min at 4°C. The suspension was incubated for 1 h at −20°C and vortexed every 15 min. It was then centrifuged at 9000 × g for 20 min at 4°C. The supernatant was discarded and the pellet was washed twice with 0.07% 2-mercaptoethanol in acetone. The final pellet was dried and re-suspended in lysis buffer, consisting of 7 M urea, 2 M thiourea, 5% 3-[(3-Cholamidopropyl) dimethylammonio]-1-propanesulfonate, and 2 mM tributylphosphine, by vortexing for 1 h at 25°C. The suspension was then centrifuged at 20,000 × g for 20 min at room temperature until a clean supernatant was obtained. The protein concentrations were determined using the Bradford method (Bradford, 1976) with bovine serum albumin as the standards.

### Protein purification and digestion for mass spectrometry analysis

Proteins (100 μg) were purified with methanol and chloroform to remove any detergent from the sample solutions. Briefly, 400 μL of methanol was added to the sample, and the resulting solution was mixed. Subsequently, 100 μL of chloroform and 300 μL of water were added to the samples, which were mixed and centrifuged at 20,000 × g for 10 min to achieve phase separation. The upper phase was discarded, and 300 μL of methanol was added slowly to lower phase. The samples were centrifuged at 20,000 × g for 10 min, supernatants were discarded, and pellets were dried. The dried pellets were resuspended in 50 mM NH_4_HCO_3_. The proteins were reduced with 50 mM dithiothreitol for 1 h at 56°C and alkylated with 50 mM iodoacetamide for 1 h at 37°C in the dark. Alkylated proteins were digested with trypsin and lysyl endopeptidase at 1:100 enzyme/protein concentrations for 16 h at 37°C. The resulting peptides were acidified with formic acid to pH < 3, and the resulting solution was centrifuged at 20,000 × g for 10 min. The obtained supernatant was collected and analyzed by nanoliquid chromatography (LC)-mass spectrometry (MS).

### Nanoliquid chromatography-tandem mass spectrometry analysis

Peptides were analyzed on a nanospray LTQ XL Orbitrap MS (Thermo Fisher Scientific, San Jose, CA, USA) operated in data-dependent acquisition mode with the installed XCalibur software (version 2.0.7, Thermo Fisher Scientific). Using an Ultimate 3000 NanoLC system (Dionex, Germering, Gemany), peptides in 0.1% formic acid were loaded onto a C18 PepMap trap column (300 μm ID × 5 mm, Dionex). The peptides were eluted from the trap column with a linear acetonitrile gradient (8–30% for 120 min) in 0.1% formic acid and at a flow rate of 200 nL·min^−1^. The peptides that were eluted from the trap column were separated on a C18 capillary tip column (75 μm ID × 120 mm, Nikkyo Technos, Tokyo, Japan) and ionized at a spray voltage of 1.5 kV. Full-scan mass spectra were acquired in the LTQ Orbitrap mass spectrometer over 400–1500 m/z with a resolution of 30,000. A lock mass function was used for high mass accuracy (Olsen et al., 2005). The 10 most intense precursor ions were selected for collision-induced fragmentation in the linear ion trap at normalized collision energy of 35%. Dynamic exclusion was employed within 90 s to prevent repetitive selection of peptides (Zhang et al., 2009).

### Protein identification from acquired mass spectrometry data

Identification of proteins was performed using the Mascot search engine (version 2.5.1; Matrix Science, London, UK) with the NCBInr-Viridiplantae database (2, 564, 738 sequences) obtained from the NCBI database (http://www.nibi.nlm.nih.gov/protein). The acquired raw data files were processed using Proteome Discoverer (version 1.4.0.288; Thermo Fisher Scientific). The parameters used in Mascot searches were as follows: carbamidomethylation of cysteine was set as a fixed modification, and oxidation of methionine was set as a variable modification. Trypsin was specified as the proteolytic enzyme and one missed cleavage was allowed. Peptide mass tolerance was set at 10 ppm, fragment mass tolerance was set at 0.8 Da, and the peptide charge was set at +2, +3, and +4. An automatic decoy database search was performed as part of the search. Mascot results were filtered with Mascot Percolator to improve the accuracy and sensitivity of peptide identification (Brosch et al., 2009). False discovery rates for peptide identification of all search strategies were < 1.0%. Peptides with a percolator ion score of more than 13 (*p* < 0.05) were used for protein identification. The acquired Mascot results were exported into the SIEVE software (version 2.1.377; Thermo Fisher Scientific) for differential analysis.

### Differential analysis of proteins using mass spectrometry data

Proteins that contained common peptides were grouped. Both unique and shared peptides were analyzed for protein quantification. Peptides that are shared between proteins were only included in the quantitative information for the protein that contained the most number of assigned peptides to ensure that a peptide was only used once for quantification. The chromatographic peaks detected by MS were aligned, and the peptide peaks were detected as frames on all parent ions scanned by MS/MS using 5 min of frame time width and 10 ppm of frame m/z width. In the differential analysis of protein abundance, total ion current was used for normalization. Chromatographic peak areas within a frame were compared for each sample, and the ratios between samples were determined for each frame. The frames detected in the MS/MS scan were matched to the imported Mascot results. The peptide ratios between samples were determined from the variance-weighted average of the ratios in frames that matched the peptides in the MS/MS spectrum. The ratios of peptides were further integrated to determine the ratios of the corresponding proteins. For identification of differentially changed proteins, the minimum requirements for the identification of differentially changed proteins were: at least six peptide sequence matches above the identity threshold with more than 10% sequence coverage; the number of matched peptides with more than two peptides; and a fold change of more or less than 1.2 times in protein quantities in the treated samples against the control with a significant difference (*p* < 0.05).

### RNA extraction and quantitative reverse transcription-polymerase chain reaction analysis

Samples (0.1 g fresh weight) were ground to powder in liquid nitrogen using a sterilized mortar and pestle. Total RNA was extracted from the tissue powder using a Quick RNA Isolation Kit (Huayueyang Biotechnology, Beijing, China). RNA was reverse-transcribed using a Reverse Transcription System (Promega, Madison, WI, USA) according to the manufacturer's protocol. Primers were designed using the Primer Premier 5.0 (Supplementary Table 1). The qRT-PCR was performed in a 10 μL reaction using a QuantiFast SYBR Green PCR Kit (Qiagen, Hilden, Germany) on an IQ5 Multicolor Real-Time PCR Detection System (Bio-Rad, Hercules, CA, USA). The relative quantification method (2^−ΔΔCT^) was used to evaluate quantitative variation between treatments. *40S Ribosomal protein S9* (RPS9) was used as a single reference gene (Menke et al., 1999).

### Functional categorization of identified proteins

Protein functions were categorized using MapMan bin codes (http://mapman.gabipd.org/), as previously described (Usadel et al., 2005). A small-scale prediction of the identified proteins from *C. roseus* was performed by transferring annotations from the *Arabidopsis* genome and consideration of orthologous genes.

### Statistical analysis

SPSS 19.0 (IBM, Chicago, IL, USA) statistical software was used for the statistical evaluation of the results. One-Way ANOVA followed by Tukey's multiple comparison post-hoc tests and the Student's *t*-test was also performed when only two groups were compared. All results were shown as mean ± SD from three independent biological replicates. A *p* value of less than 0.05 was considered statistically significant.

## Results

### The contents of indole alkaloids in *C. roseus* leaf increased under binary stress

**Figure 1:**
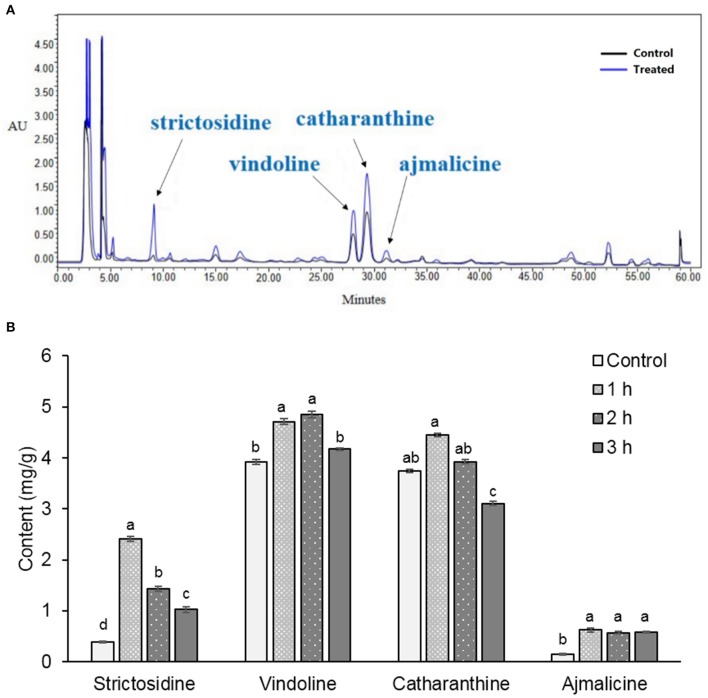
**Pattern of metabolites in leaves of *C. roseus.*** Methanol extracts from leaves collected from 45-day-old *C. roseus* plants were analyzed by HPLC **(A)**. Arrowheads show the positions of the identified alkaloids. Control: *C. roseus* plants were only incubated for 72 h in the dark (black line). Treated: *C. roseus* plants were irradiated by UV-B for 1 h and incubated for 72 h in the dark (blue line). The content of the alkaloids in *C. roseus* under 1, 2, and 3 h UV-B irradiation times were calculated from standard curves **(B)**. Data were shown as means ± SD from three independent biological replicates. Means with the same letter are not significantly different according to One-Way ANOVA test (*p* < 0.05). Control, plants with only 72 h of dark incubation.

To profile changes in indole alkaloids biosynthesis in *C. roseus* plant under stresses, the indole alkaloids in *C. roseus* leaves were analyzed. Methanol extracts of *C. roseus* leaves were examined by HPLC analysis. Based on retention times and ultraviolet absorbance of the standards, the four alkaloids containing strictosidine, ajmalicine, vindoline, and catharanthine were identified in leaves. The contents of alkaloids in *C. roseus* leaves increased after 1 h of UV-B irradiation followed by 72 h of dark incubation (Figure 1A). Time-course quantitative analysis of the induced alkaloids demonstrated that 1 h of UV-B irradiation coupled with 72-h of dark incubation were the optimal induction conditions, under which the total contents of the four compounds were at maximum (Figure 1B). Compared with the control, the contents of strictosidine, ajmalicine, vindoline, and catharanthine in *C. roseus* leaves after 1 h of UV-B irradiation and 72 h of dark incubation increased by 527.9, 321.6, 20.1, and 19.0%, respectively (Supplementary Table 2).

### Quantitative proteomic analysis reveals identities of proteins that were responsive to binary stress

**Figure 2:**
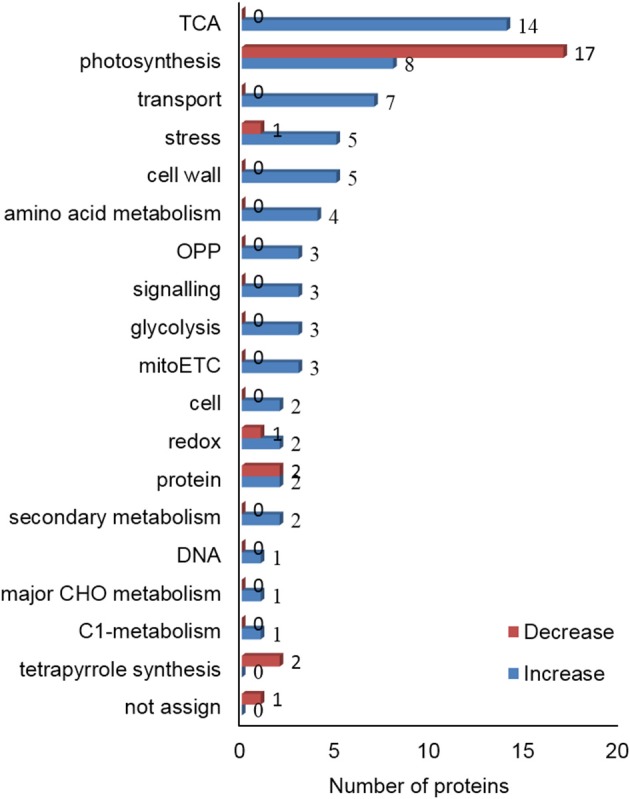
**Functional categorization of differential proteins in leaves of *C. roseus* treated with UV-B and dark stress**. *C. roseus* plants were irradiated with or without 1 h of UV-B irradiation and 72 h of dark incubation. Leaves were collected and proteins were then extracted, digested and analyzed by nanoLC-MS/MS. The identified proteins were categorized using MapMan bin codes. Numbers of categorized proteins are shown in the graph. Blue bars and red bars indicate increased and decreased levels of proteins. The abbreviations are as follows: TCA, tricarboxylic acid cycle; OPP, oxidative pentose phosphate; mitoETC, mitochondrial electron transport chain; cell, cell organization/vesicle transport; redox, redox ascorbate/glutathione metabolism; protein, protein synthesis/posttranslational modification/folding/degradation/activation; DNA, DNA synthesis/repair; CHO, carbohydrates; and C1-metabolism, carbon 1-metabolism.

Gel-free proteomic technique was employed to reveal changes in protein abundance that accounted for the increase in alkaloid contents in *C. roseus* leaves. The leaves that were treated with or without 1 h of UV-B irradiation followed by 72 h of dark incubation were collected. Proteins were extracted, reduced, alkylated, and digested. The tryptic peptides were collected. Principle component analysis of the peptide profile for total proteins was performed for quality assessment of the experimental inputs (Supplementary Figure 2). A total of 90 proteins with more than 2 matched peptides were identified as being differentially changed (fold change > 1.2 and *p* < 0.05). Out of these 90 proteins, the accumulation level of 24 proteins was decreased and that of 66 proteins was increased significantly in C. roseus leaves upon UV-B irradiation and dark incubation (Table 1, Supplementary Table 3). These differential proteins were categorized into 18 groups (Figure 2). The abundance of proteins related to oxidative pentose phosphate, tricarboxylic acid (TCA) cycle, cell wall, glycolysis, transport, amino acid metabolism, secondary metabolism, C1-metabolism, major CHO metabolism, mitochondrial electron transport, and signaling were increased after UV-B irradiation and dark incubation. In contrast, the abundance of proteins related to tetrapyrrole synthesis were decreased. The abundance of proteins related to photosynthesis, redox ascorbate/glutathione metabolism, stress, and protein synthesis followed either an increasing or decreasing trend. Additionally, the abundance of 10-hydroxygeraniol oxidoreductase (10-HGO) in *C. roseus* leaves, which is involved in the pathway of indole alkaloid biosynthesis, was 2 times higher than that of the control group.

**Table 1:** **Total proteins changed between control and UV-B treatment by gel-free proteomics**.

**Protein ID**	**Ortholog^a^**	**Description**	**M.P.^b^**	**Ratio^c^**	**Functional category^d^**
**INCREASE**					
GI:30693656	At3g52560	Ubiquitin-conjugating enzyme E2 variant 1D [*Arabidopsis thaliana*]	2	2.16	Protein
GI:159471081	At3g02230	UDP-Glucose:protein transglucosylase [*Chlamydomonas reinhardtii*]	2	2.03	Cell wall
GI:33519154	–	10-hydroxygeraniol oxidoreductase [*Camptotheca acuminata*]	2	1.98	Secondary metabolism
GI:357471289	At-105710	Aconitate hydratase [*Medicago truncatula*]	2	1.96	TCA
GI:21536725	At5g41670	6-phosphogluconate dehydrogenase [*Arabidopsis thaliana*]	4	1.94	OPP
GI:448872690	At3g02230	Alpha-1,4-glucan-protein synthase [*Elaeis guineensis*]	3	1.93	Cell wall
GI:402228002	At4g37990	Cinnamyl alcohol dehydrogenase [Fragaria × ananassa]	2	1.88	Secondary metabolism
GI:508706435	At5g09590	Mitochondrial HSO70 2 isoform 2 [*Theobroma cacao*]	2	1.87	Stress
GI:525507286	At3g52880	Monodehydroascorbate reductase, seedling isozyme [*Cucumis sativus*]	2	1.86	Redox
GI:195633817	At4g21280	Oxygen-evolving enhancer protein 3-1 [Zea mays]	2	1.83	Photosynthesis
GI:473888752	At5g03290	Isocitrate dehydrogenase [NAD] catalytic subunit 5 [Triticum urartu]	2	1.82	TCA
GI:297807587	At5g15650	Reversibly glycosylated polypeptide_3 [*Arabidopsis lyrata* subsp. lyrata]	2	1.77	Cell wall
GI:351727547	At3g61440	OAS-TL3 cysteine synthase [Glycine max]	3	1.77	Amino acid metabolism
GI:162463414	At5g15650	Alpha-1,4-glucan-protein synthase [UDP-forming] [Zea mays]	2	1.76	Cell wall
GI:297802428	At4g35220	Cyclase family protein [*Arabidopsis lyrata* subsp. lyrata]	2	1.74	DNA
GI:351722265	At3g02360	6-phosphogluconate dehydrogenase [Glycine max]	2	1.73	OPP
GI:213493066	At1g65930	NADP-dependent isocitrate dehydrogenase [Passiflora edulis]	2	1.72	TCA
GI:285309965	At4g35830	Aconitate hydratase 1 [Citrus clementina]	3	1.72	TCA
GI:213959111	At3g12580	Heat shock protein 70 [*Oryza sativa* Japonica Group]	2	1.72	Stress
GI:508707706	At5g13420	Aldolase-type TIM barrel family protein isoform 2, partial [*Theobroma cacao*]	2	1.72	OPP
GI:357491797	At1g65930	Isocitrate dehydrogenase [*Medicago truncatula*]	2	1.63	TCA
GI:18414289	At4g14710	1,2-dihydroxy-3-keto-5-methylthiopentene dioxygenase 3 [*Arabidopsis thaliana*]	2	1.63	Amino acid metabolism
GI:285309969	At2g05710	Aconitate hydratase 2 [Citrus clementina]	2	1.62	TCA
GI:475618083	At5g15650	Alpha-1,4-glucan-protein synthase (UDP-forming) [*Aegilops tauschii*]	3	1.61	Cell wall
GI:197717740	At1g09210	Calreticulin [*Nicotiana tabacum*]	2	1.59	Signaling
GI:297800722	At4g14880	O_acetylserine (thiol) lyase (oas_tl) isoform A1 [*Arabidopsis lyrata*]	2	1.55	Amino acid metabolism
GI:508778858	At3g12580	Heat shock protein 70B [*Theobroma cacao*]	2	1.53	Stress
GI:15218869	At1g65930	Isocitrate dehydrogenase [*Arabidopsis thaliana*]	3	1.51	TCA
GI:315440254	At3g60100	Mitochondrial citrate synthase [*Pyrus pyrifolia*]	4	1.51	TCA
GI:285309967	At4g26970	aconitate hydratase 3 [Citrus clementina]	3	1.46	TCA
GI:474119554	At4g38510	V-type proton ATPase subunit B 1 [Triticum urartu]	2	1.45	Transport
GI:357439825	At4g13430	3-isopropylmalate dehydratase [*Medicago truncatula*]	2	1.44	TCA
GI:357445271	At5g49910	Heat shock protein [*Medicago truncatula*]	2	1.43	Stress
GI:351726325	At3g09640	L-ascorbate peroxidase 2 [Glycine max]	2	1.43	Redox
GI:308808864	At1g78900	Vacuolar H__ATPase V1 sector_ subunit A (ISS) [*Ostreococcus tauri*]	2	1.42	Transport
GI:390098820	At2g11270	Citrate synthase [*Capsicum annuum*]	2	1.41	TCA
GI:37903393	At4g09000	14-3-3-like protein [Saccharum hybrid cultivar CP65-357]	2	1.41	Signaling
GI:381145559	At3g53230	cell division cycle protein 48 [*Camellia sinensis*]	2	1.4	Cell
GI:297819360	At5g01530	chlorophyll a/b_binding protein [*Arabidopsis lyrata* subsp. lyrata]	2	1.38	Photosynthesis
GI:374433978	At3g14420	glycolate oxidase [Wolffia australiana]	3	1.38	Photosynthesis
GI:18409740	At3g52990	pyruvate kinase [*Arabidopsis thaliana*]	2	1.38	Glycolysis
GI:214010947	At2g13360	serine glyoxylate aminotransferase 3 [Glycine max]	2	1.37	Photosynthesis
GI:308812360	At3g12110	ACT_COLSC Actin (ISS) [*Ostreococcus tauri*]	2	1.37	Cell
GI:7547401	At5g40810	cytochrome c1 precursor [*Solanum tuberosum*]	2	1.37	mitoETC
GI:302850070	At2g07698	F1F0 ATP synthase_ subunit alpha_ mitochondrial [*Volvox carteri* f. nagariensis]	2	1.37	mitoETC
GI:15231937	At3g08580	ADP,ATP carrier protein 1 [*Arabidopsis thaliana*]	2	1.34	Transport
GI:297807205	At5g11670	NADP_malic enzyme 2 [*Arabidopsis lyrata* subsp. lyrata]	2	1.32	TCA
GI:508710478	At5g14780	Formate dehydrogenase [*Theobroma cacao*]	2	1.32	C1-metabolism
GI:302127766	At1g53310	C3 phosphoenolpyruvate carboxylase [Panicum bisulcatum]	3	1.32	Glycolysis
GI:42416979	At4g33010	glycine dehydrogenase P protein [*Oryza sativa* Indica Group]	4	1.31	Photosynthesis
GI:474012573	At5g02500	Heat shock cognate 70 kDa protein 1 [Triticum urartu]	4	1.3	Stress
GI:940877	At5g14590	Isocitrate dehydrogenase (NADP+) [*Solanum tuberosum*]	2	1.29	TCA
GI:195605636	At3g55440	triosephosphate isomerase, cytosolic [Zea mays]	2	1.29	Glycolysis
GI:15229784	At3g43810	calmodulin 7 [*Arabidopsis thaliana*]	2	1.28	Signaling
GI:15226854	At2g02010	glutamate decarboxylase 4 [*Arabidopsis thaliana*]	2	1.27	Amino acid metabolism
GI:4239891	At1g79750	NADP-malic enzyme [*Aloe arborescens*]	2	1.26	TCA
GI:473968259	At1g32060	Phosphoribulokinase, chloroplastic [Triticum urartu]	2	1.26	Photosynthesis
GI:226500740	At2g19860	hexokinase-1 [Zea mays]	2	1.26	Major CHO metabolism
GI:162134229	AtMg01190	ATP synthase F1 subunit alpha [Trebouxia aggregata]	2	1.25	mitoETC
GI:15219234	At1g78900	V-type proton ATPase catalytic subunit A [*Arabidopsis thaliana*]	7	1.24	Transport
GI:15233115	At3g54890	light-harvesting complex I chlorophyll a/b binding protein 1 [*Arabidopsis thaliana*]	3	1.23	Photosynthesis
GI:297812157	At5g19760	dicarboxylate/tricarboxylate carrier [*Arabidopsis lyrata* subsp. lyrata]	2	1.23	Transport
GI:195658441	At1g78900	vacuolar ATP synthase catalytic subunit A [Zea mays]	3	1.23	Transport
GI:240254125	At1g20260	V-type proton ATPase subunit B3 [*Arabidopsis thaliana*]	5	1.23	Transport
GI:474369193	At1g23490	ADP-ribosylation factor 2 [Triticum urartu]	2	1.22	Protein
GI:224127848	At4g10340	light-harvesting complex II protein Lhcb5 [*Populus trichocarpa*]	2	1.21	Photosynthesis
**DECREASE**					
GI:18411711	At3g60750	transketolase [*Arabidopsis thaliana*]	2	0.83	Photosynthesis
GI:297794397	At5g66190	ferredoxin_NADP_ reductase [*Arabidopsis lyrata* subsp. lyrata]	2	0.82	Photosynthesis
GI:78675163	AtCg00270	photosystem II protein D2 [Lactuca sativa]	2	0.82	Photosynthesis
GI:307136095	At5g66190	ferredoxin–NADP reductase [*Cucumis melo* subsp. melo]	3	0.82	Photosynthesis
GI:297840973	AtCg00340	photosystem I P700 chlorophyll a apoprotein A2 [*Arabidopsis lyrata* subsp. lyrata]	2	0.81	Photosynthesis
GI:511348322	AtCg00130	ATP synthase CF0 subunit I (chloroplast) [*Catharanthus roseus*]	2	0.81	Photosynthesis
GI:27448410	AtCg00490	ribulose-1,5-bisphosphate carboxylase/oxygenase large subunit [Selaginella wrightii]	2	0.79	Photosynthesis
GI:374975197	AtCg00490	ribulose-1,5-bisphosphate carboxylase/oxygenase large subunit [*Neoregelia carolinae*]	4	0.79	Photosynthesis
GI:413968524	At1g31330	photosystem I subunit III precursor (chloroplast) [*Solanum tuberosum*]	2	0.77	Photosynthesis
GI:89280643	AtCg00490	ribulose-1,5-bisphosphate carboxylase/oxygenase large subunit [*Solanum lycopersicum*]	2	0.76	Photosynthesis
GI:428697696	AtCg00490	ribulose 1_5_bisphosphate carboxylase/oxygenase large subunit [Pellia endiviifolia]	2	0.76	Photosynthesis
GI:443329645	AtCg00490	ribulose 1_5_bisphosphate carboxylase/oxygenase large subunit [*Equisetum hyemale*]	3	0.76	Photosynthesis
GI:18412632	At4g04640	ATP synthase gamma chain 1 [*Arabidopsis thaliana*]	2	0.75	Photosynthesis
GI:224179477	AtCg00490	ribulose_1_5_bisphosphate carboxylase/oxygenase large subunit [Monomastix sp.]	4	0.75	Photosynthesis
GI:139389650	AtCg00490	large subunit of riblose-1,5-bisphosphate carboxylase/oxygenase [*Arabis hirsuta*]	3	0.7	Photosynthesis
GI:508781449	At4g04640	ATPase, F1 complex, gamma subunit protein [*Theobroma cacao*]	2	0.66	Photosynthesis
GI:508777181	At1g62750	Translation elongation factor EFG/EF2 protein [*Theobroma cacao*]	2	0.57	Protein
GI:474036467	At5g45930	Magnesium-chelatase subunit chlI, chloroplastic [Triticum urartu]	3	0.55	Tetrapyrrole synthesis
GI:148763638	At5g45930	Magnesium chelatase 40-kDa subunit [*Hordeum vulgare* subsp. vulgare]	2	0.52	Tetrapyrrole synthesis
GI:297804102	At4g20360	Chloroplast elongation factor tub [*Arabidopsis lyrata* subsp. lyrata]	2	0.42	Protein
GI:300681420	At3g55800	sedoheptulose-1,7-bisphosphatase, chloroplast precursor, expressed [*Triticum aestivum*]	2	0.36	Photosynthesis

a Ortholog, indicates AGI E.C.code.

b M.P., indicates number of matched peptide, the proteins with >2 matched peptides and p-value < 0.05 were considered.

c Ratio, indicates ratio of quantities of protein (UV-B treated group/control group), the proteins with ratio 1.2/0.8 were considered.

d “Functional category” indicates protein function categorized using MapMan bin codes.

### Expression of genes involved in the biosynthesis of indole alkaloid in *C. roseus* was up-regulated in response to binary stress

**Figure 3:**
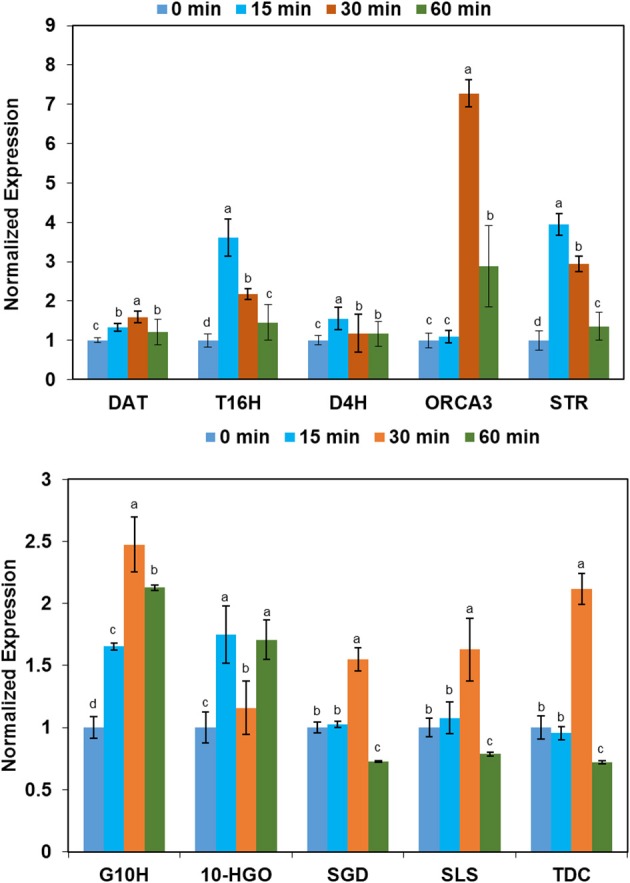
**The variation in expression tendency of the selected genes of leaves derived from *C. roseus* under conditions of different UV-B exposure**. The leaves of *C. roseus* plants were collected at 0, 15, 30, and 60 min UV-B irradiation. Total RNA was extracted from the collected leaves. The transcript abundance of the selected genes was analyzed by qRT-PCR. Data were shown as means ± SD from three independent biological replicates. Means with the same letter are not significantly different according to One-Way ANOVA test (*p* < 0.05). The abbreviations are follows: the genes *tdc, g10h,10-hgo, sls, str, sgd, t16h, d4h, dat*, and *ORCA3* code for tryptophan decarboxylase,geraniol-10-hydroxylase, 10-hydroxygeraniol oxidoreductase, secologanin synthase, strictosidine synthase, strictosidine β-glucosidase, tabersonine 16-hydroxylase, deacetoxyvindoline 4-hydroxylase, 6-17-O-deacetylvindoline O-acetyltransferase and Octadecaniod-derivative Responsive Catharanthus AP2-domain Protein 3, respectively. The *RPS9* gene was used as a reference control gene.

To uncover the molecular mechanism of increased indole alkaloid in *C. roseus* upon exposure to binary stress, qRT-PCR analysis was performed. Forty five-day-old *C. roseus* were treated with UV-B irradiation for 0, 15, 30, and 60 min and RNAs extracted from leaves were analyzed by qRT-PCR. The nine genes and one transcription factor containing *tryptophan decarboxylase (tdc), geraniol-10-hydroxylase (g10h), 10-hydroxygeraniol oxidoreductase (10hgo), secologanin synthase (sls), strictosidine synthase (str), strictosidine* β*-glucosidase (sgd), tabersonine 16-hydroxylase (t16h), deacetoxyvindoline 4-hydroxylase (d4h), 6-17-O-deacetylvindoline O-acetyltransferase (dat)*, and *Octadecaniod-derivative Responsive Catharanthus AP2-domain Protein 3 (ORCA3)*, which are involved in the biosynthesis pathway of the alkaloids, were selected for further analysis of mRNA expression levels (Supplementary Figure 3). *RPS9* was selected as a reference gene (Supplementary Table 1). Among the examined genes, the mRNA expression levels of *dat, t16h, d4h, ORCA3, str, g10h*, and *10-hgo* were up-regulated in leaves under UV-B stress. In addition, the expression levels of *t16h, ORCA3*, and *str* in *C. roseus* leaf after UV-B irradiation were four times higher than those of the control. The levels of *sgd, sls*, and *tdc* were up-regulated after 30-min of UV-B irradiation, but down-regulated after 60 min of UV-B irradiation (Figure 3).

### The physiological activity of *C. roseus* was changed under binary stress

Physiological parameters were determined to reflect the physiological activities of *C. roseus* leaves after UV-B irradiation and dark incubation. The leaves of *C. roseus* plants that were treated with or without 1 h of UV-B irradiation and 72 h of dark incubation were collected and homogenized in buffer solution. After centrifugation, the supernatants were collected for the analysis of physiological parameters. After UV-B irradiation and dark incubation, the contents of soluble protein, soluble sugar, MDA, and proline were decreased by 5.6, 7.5, 24, and 19.2%, respectively; while, the content of glutathione was increased by 33%. Compared with the control, the activities of SOD and POD were increased by 21.4 and 124%, respectively. In contrast, the activity of glutathione reductase was decreased by 31.4% (Table 2). However, the content of photosynthetic pigments did not show a significant change after UV-B irradiation and dark incubation (Supplementary Table 4).

**Table 2:** **Changes of physiological parameters in *C. roseus* leaves**.

	**Soluble sugar (mg/g)**	**Soluble protein (mg/g)**	**SOD [U/mg(pro)]**	**GR^a^ [U/g(pro)]**	**POD [U/(g.min)]**	**MDA (umol/g)**	**Proline (ug/g)**	**GSH^b^(mg/g)**
Control	5.288 ± 0.067	8.517 ± 0.058	122.361 ± 2.465	39.360 ± 2.622	1255.093 ± 20.789	0.0071 ± 0.0001	35.880 ± 1.363	0.058 ± 0.002
Treated	4.990 ± 0.027^*^	7.879 ± 0.167^*^	148.501 ± 1.671^*^	27.009 ± 2.462^*^	2810.691 ± 46.463^*^	0.0054 ± 0.0004^*^	29.005 ± 1.373^*^	0.077 ± 0.003^*^

* Significant at P < 0.05;

a GR, glutathione reductase;

b GSH, glutathione.

## Discussion

### The increase in indole alkaloid biosynthesis in *C. roseus* is induced by binary stress

Although UV-B serves as an adverse environmental factor for the growth and development of plants (Caldwell et al., 2007), it has been shown to induce a variety of bioactive secondary metabolites, such as indole alkaloids, in medicinal plants (Ramani and Chelliah, 2007). Indole alkaloids provide protection against microbial infection, herbivores consumption, and abiotic environmental stresses (Cordell, 2013). In this study, the contents of the indole alkaloids strictosidine, vindoline, catharanthine, and ajmalicine in *C. roseus* leaves were increased under the binary stress of UV-B irradiation and dark incubation. Both UV-B irradiation and dark incubation are essential stresses for alkaloid induction (Zhang et al., 2014). Additionally, it is known that reactive oxygen species (ROS) are largely released under conditions of high-energy irradiation of UV-B, resulting in oxidative injury and disturbance of metabolism in plants (Apel and Hirt, 2004). It is likely that the alkaloids are accumulated to enhance the scavenging capacity of ROS. Moreover, the enhanced activities of the enzymatic and non-enzymatic antioxidant systems also indicate the accumulation of ROS.

The accumulation of 10-HGO, which is a key enzyme involved in the alkaloid biosynthesis pathway, provides further insights into the induction of the indole alkaloids. 10-HGO also plays a key role in the secoiridoid pathway and catalyzes the synthesis of secologanin which is one of the substrates for strictosidine synthase (Valletta et al., 2010). The other substrate for strictosidine synthase is tryptamine, which is derived from tryptophan metabolism via the shikimate pathway. Strictosidine synthase catalyzes the condensation of secologanin and tryptamine to yield strictosidine, which is the key precursor for the biosynthesis of indole alkaloids in *C. roseus* (Yamazaki et al., 2003). In the present study, 10-HGO was two-fold more abundant in the treated group compared to the control group, which may explain the increased amount of strictosidine observed after stress treatment.

To verify the mechanism behind the change in the alkaloid at the transcript level, 10 key genes, which are involved in indole alkaloids biosynthesis, were selected for investigation by qRT-PCR. The genes *tdc, g10h, 10-hgo, sls*, and *str* encode the key enzymes that catalyze the biosynthesis of strictosidine from tryptophan and geraniol, which is a key intermediate in indole alkaloid biosynthesis (Patra et al., 2013). *ORCA3* is a transcription factor that plays a key role in controlling several important key genes involved in indole alkaloid biosynthesis (Goklany et al., 2013). The overexpression of *ORCA3* increases the expression of indole alkaloid biosynthetic genes, leading to the accumulation of indole alkaloids (van der Fits and Memelink, 2000). In the present study, the continual up-regulated expression of *tdc, g10h, 10-hgo, sls*, and *str* was consistent with the increased content of strictosidine in *C. roseus* under stress. Similarly, the up-regulated expression of *sgd, d4h, dat, ORCA3*, and *t16h* may have contributed to the observed increasing contents of ajmalicine, vindoline, and catharanthine under binary stress. Furthermore, the variation tendency in gene expression under stresses was the same as that of alkaloids. Taken together, our results indicate that the genes involved in the biosynthesis of indole alkaloids are induced under binary stress, which may account for the increased contents of indole alkaloids in *C. roseus*.

### Enhanced tricarboxylic acid cycle in *C. roseus* might be related to the biosynthesis of secondary metabolites

The abundance of aconitate hydratase, iscocitrate dehydrogenase, and mitochondrial citrate synthase which were involved in the TCA cycle, was increased in *C. roseus* under UV-B irradiation and dark incubation. The TCA cycle which is composed of a set of eight enzymes can oxidize pyruvate and malate to form CO_2_ and nicotinamide adenine dinucleotide (NADH) and provide intermediates for metabolic pathways (Nunes-Nesi et al., 2013). Aconitase hydratase which is involved in the TCA cycle plays a vital role in nutrient metabolism and energy supply (Zhang et al., 2003). NADP dependent iscocitrate dehydrogenase is related to the synthesis of 2-oxoglutarate for ammonia assimilation and glutamate in plants (Palomo et al., 1998). Additionally, citrate synthase catalyzes the condensation reaction to form citrate (Mulholland and Richards, 1997). Functioning with the described key enzymes, the TCA cycle provides ATP for the fundamental physiological activities of plants (Fernie et al., 2004). In *C. roseus* leaves, the enhanced TCA cycle may serve to provide energy and intermediates for secondary metabolism. Furthermore, the increase in abundance of proteins related to primary metabolism, such as acetylserine lyase, cysteine synthase, and aldolase-type barrel family protein isoforms, and the resulting accumulation of the primary metabolites, might promote the biosynthesis of secondary metabolites.

### Fundamental physiology of *C. roseus* was influenced by binary stress

UV-B can induce plant photomorphogenic development which is characterized by the inhibition of flavonoid accumulation and UV-B stress tolerance (Huang et al., 2014). Low-intensity UV-B radiation can act as entraining signal for the circadian clock (Feher et al., 2011). Photomorphogenic UV-B signaling also controls plant immune response (Demkura and Ballare, 2012). Photosynthesis has been reported as a direct and most affected system in plants upon UV-B stress, which frequently results in reduced abundance of photosynthesis related proteins (Strid et al., 1994; Kanalas et al., 2008). In the present study, the abundance of proteins related to photosynthesis such as sedoheptulose-1, 7-bisphosphatase, chlorophyll a/b binding protein, serine glyoxylate aminotransferase 3, ribulose bisphosphate carboxylase/oxygenase, and light-harvesting complex II protein were decreased in *C. roseus* leaves. It is known that chlorophyll can be synthesized via a common tetrapyrrole biosynthetic pathway. In our study, the abundance of two tetrapyrrole synthesis proteins were also decreased after UV stress. Moreover, this observation was consistent with the content of the photosynthetic pigment observed in *C. roseus* leaves. This evidence suggests that binary stress might negatively affect the process of photosynthesis in *C. roseus.*

Cinnamyl alcohol dehydrogenases is a zinc-dependent dehydrogenase, responsible for catalyzing the reversible conversion of *p*-hydroxycinnamaldehydes to the corresponding alcohols in lignin biosynthesis (Blanco-Portales et al., 2002). Lignin is one of the most important components in plant cell walls, which protects cellulose in the cell walls of plants by adhering to hydrolytic enzymes (Moura et al., 2010). UDP-glucose: protein transglucosylase (UPTG) catalyzes the synthesis of α-1, 4-glucan, which is related to starch biosynthesis and plays a crucial role in the synthesis of cell wall polysaccharides (Bocca et al., 1999). In addition it has been shown to relate to cell wall compound metabolism and contribute to cell wall thickening (Ge et al., 2007). In this study, the *E. guineensis* and in *Z. mays* orthologs of alpha-1,4-glucan-protein synthase were identified (Supplementary Table 3). The abundance of cinnamyl alcohol dehydrogenases and proteins related to cell wall such as alpha-1,4-glucan-protein synthase, reversibly glycosylated polypeptide, and UPTG were increased compared to the control group. These results suggest that the increase of cell wall formation is a response to binary stress in *C. roseus*.

Furthermore, heat shock proteins (HSP) are produced by cells in response to environmental stress conditions in plants (Wang et al., 2004). HSP-70 is widely distributed in plants and is responsible for preventing aggregation and assisting refolding of non-native proteins under stressful conditions (Frydman, 2001). Some studies found that HSP 70 participates in photo-protection and the process of photosystem II repair (Schroda et al., 1999). The up-regulated Hsp70 level may be involved in the modulation of signal transduction and maintenance of key functions of basic cellular proteins after UV-B exposure and dark stress conditions such as damaged photosystem II centers from UV-B irradiation.

## Concluding remarks

In conclusion, our data indicated that the increase of indole alkaloids production in *C. roseus* leaves under binary stress was a consequence of metabolic pathways alteration. Comparative gel-free proteomics revealed significant proteome difference in *C. roseus* leaves between control and treated groups. The proteins related to cell wall and secondary metabolism in *C. roseus* leaves might be parts of the defense system against injuries resulted from binary stress. The alteration in secondary metabolism, might account for the increase in indole alkaloids observed in *C. roseus* leaves after stress exposure. In addition, the enhanced TCA cycle might affect the biosynthesis of indole alkaloid in *C. roseus*. Finally, the data derived from this study is highly applicable to other medicinal plant and provides important insights for effectively improving the contents of alkaloids in medicinal plants for industrial production.

## Accession codes

The mass spectrometry proteomics data have been deposited with the ProteomeXchange Consortium (http://proteomecentral.proteomexchange.org) via the PRIDE partner repository (Vizcaino et al., 2013) with the dataset identifier PXD001937.

### Conflict of interest statement

The authors declare that the research was conducted in the absence of any commercial or financial relationships that could be construed as a potential conflict of interest.

## Abbreviations

HPLC: high performance liquid chromatography
MDA: Malondialdehyde
POD: peroxidase
qRT-PCR: quantitative reverse transcription-polymerase chain reaction
ROS: reactive oxygen species
SOD: superoxide dismutase
TCA: tricarboxylic acid
10-HGO: 10-hydroxygeraniol oxidoreductase.
