# Supplementary material for: Binary stress induces an increase in indole alkaloid biosynthesis in Catharanthus roseus
Source: Front Plant Sci. 2015 Jul 28;6:582. doi: 10.3389/fpls.2015.00582 (PMC4516820; doi:10.3389/fpls.2015.00582)
Supplement: Supplementary file 2 [file DataSheet1.PDF]

Supplementary Material

Binary Stress Induces an Increase in Indole Alkaloid Biosynthesis in *Catharanthus roseus*

Wei Zhu[1], Bingxian Yang[1], Setsuko Komatsu[2], Xiaoping Lu[1], Ximin Li[1], Jingkui Tian [1, 3, *]

[1] College of Biomedical Engineering and Instrument Science, Zhejiang University, Hangzhou, HZ, China

[2] National Institute of Crop Science, National Agriculture and Food Research Organization, Tsukuba, Japan

[3] Education Ministry Key Laboratory for Biomedical Engineering, Zhejiang University, Hangzhou, HZ, China

*** Correspondence:** Dr Jingkui Tian, Zhouyi Qing Building, Room 108, College of Biomedical Engineering and Instrument Science, Zhejiang University, Zheda Road No.38, Hangzhou, 310027, China.

Email tjk@zju.edu.cn

1.1. Supplementary Tables

Supplemental Table 1. List of primers used for qRT-PCR experiments

Gene	Forward primer (5' to 3')	Reverse primer (5' to 3')
tdc	TCCGAAAACAAGCCCATCGT	AAGGAGCGGTTTCGGGGATA
g10h	TGAATGCTTGGGCAATTGGA	GCAAATTCTTCGGCCAGCAC
sls	GTTCCTTCTCACCGGAGTTG	CCCATTTGGTCAACATGTCA
str	ACCATTGTGTGGGAGGACAT	ATTTGAATGGCACTCCTTGC
sgd	TCACAAAGCTGCTGTGGAAG	CACCCGTTGTTAATGGCTCT
orca3	CGAATTCAATGGCGGAAAGC	CCTTATCTCCGCCGCGAACT
t16h	AGGACCTTGTTGATGTGCTAC	CATTGCCCAATCGACTGTTG
d4h	TACCCTGCATGCCCTCAACC	TTGAAGGCCGCCAATTTGAT
dat	GACCTAGTCCTTCCCAAACG	CCTCCATCAGCAACTTTGTG
rps9	GAGGGCCAAAACAAACTTGA	CCCTTATGTGCCTTTGCCTA

Supplemental Table 2. Quantitative analysis of alkaloid contents in *C. roseus* under different UV-B irradiation times with 72 h dark incubation

Group*	Strictosidine (mg/g)	Vindoline (mg/g)	Catharanthine (mg/g)	Ajmalicine (mg/g)
Control	0.384±0.016	3.924±0.046	3.738±0.034	0.148±0.022
1	2.411±0.050	4.713±0.058	4.448±0.030	0.624±0.040
2	1.428±0.050	4.849±0.070	3.926±0.044	0.571±0.028
3	1.027±0.054	4.174±0.022	3.103±0.038	0.582±0.022

* Control: no UV-B irradiation and 72 h dark incubation; 1: 1 h UV-B irradiation and 72 h dark incubation; 2: 2 h UV-B irradiation and 72 h dark incubation; 3: 4 h UV-B irradiation and 72 h dark incubation

Supplemental Table 4. Contents of photosynthesis pigment between control and treated group

	Chla (mg/g)	Chlb (mg/g)	Car (mg/g)	Tchl (mg/g)	Rchl (mg/g)
Control	1.031±0.030	0.380±0.017	0.173±0.002	1.411±0.047	2.711±0.041
Treated	1.071±0.008	0.401±0.010	0.171±0.001	1.472±0.017	2.669±0.056

1.2. Supplementary Figures

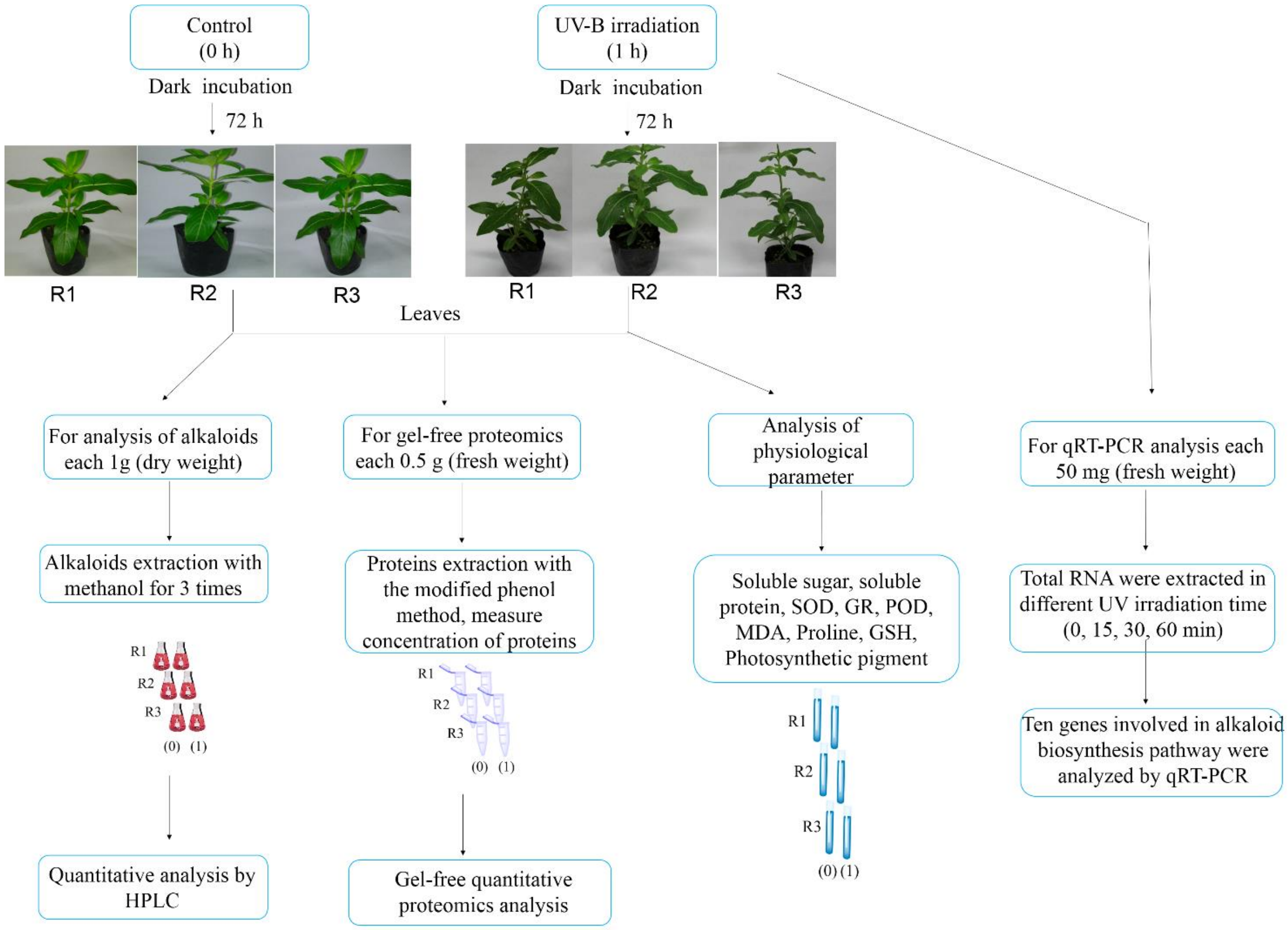

Supplementary Figure 1. Experimental design of this proteomic study. The *C. roseus* plantlets were treated with UV-B irradiation for 1 h and dark incubation for 72 h. Leaves were collected, and proteins, alkaloids, total RNA were extracted from leaves respectively. And the content of alkaloid was analyzed by HPLC. The expression of key genes in the alkaloid biosynthesis pathway were analyzed by qRT-PCR. The proteins were used for gel-free analyses. Three independent experiments were performed.

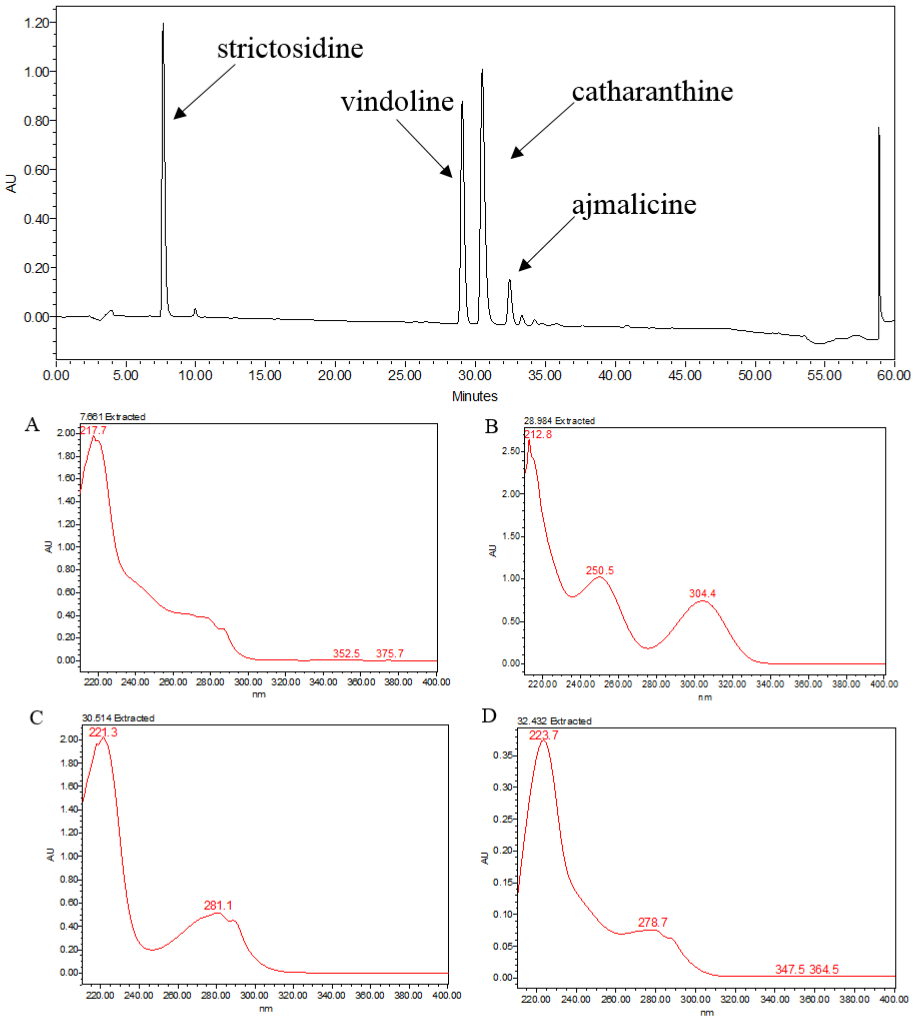

Supplementary Figure 2. HPLC chromatogram of the standards. The standards of strictosidine, vindoline, catharanthine, and ajmalicine were dissolved in methanol and diluted at six different concentrations to make calibration curve. Peaks were measured at 220 nm. (A) UV spectra with strictosidine; (B) UV spectra with vindoline; (C) UV spectra with catharanthine; (D) UV spectra with ajmalicine.

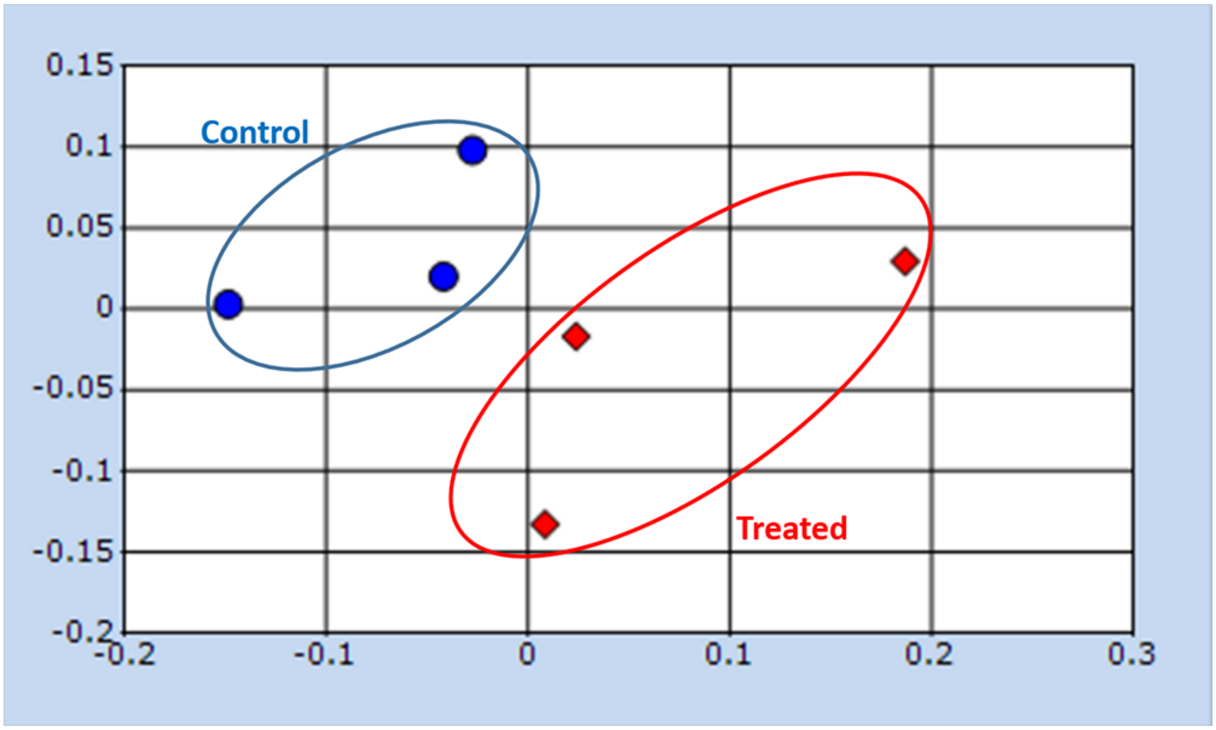

Supplementary Figure 3. Principle component analysis of the peptide profile of total proteins. The datasets of *C. roseus* proteins not exposed to UV-B irradiation plus 72 h of dark incubation, and following 1 h of UV-B irradiation plus 72 h of dark incubation were plotted using PCA. PCA was fully integrated into the SIEVE software. The blue plot was a control group which was treated only by 72 h of dark incubation. The red plot was a treated group, which was irradiated by UV-B for 1h and 72 h of dark incubation.

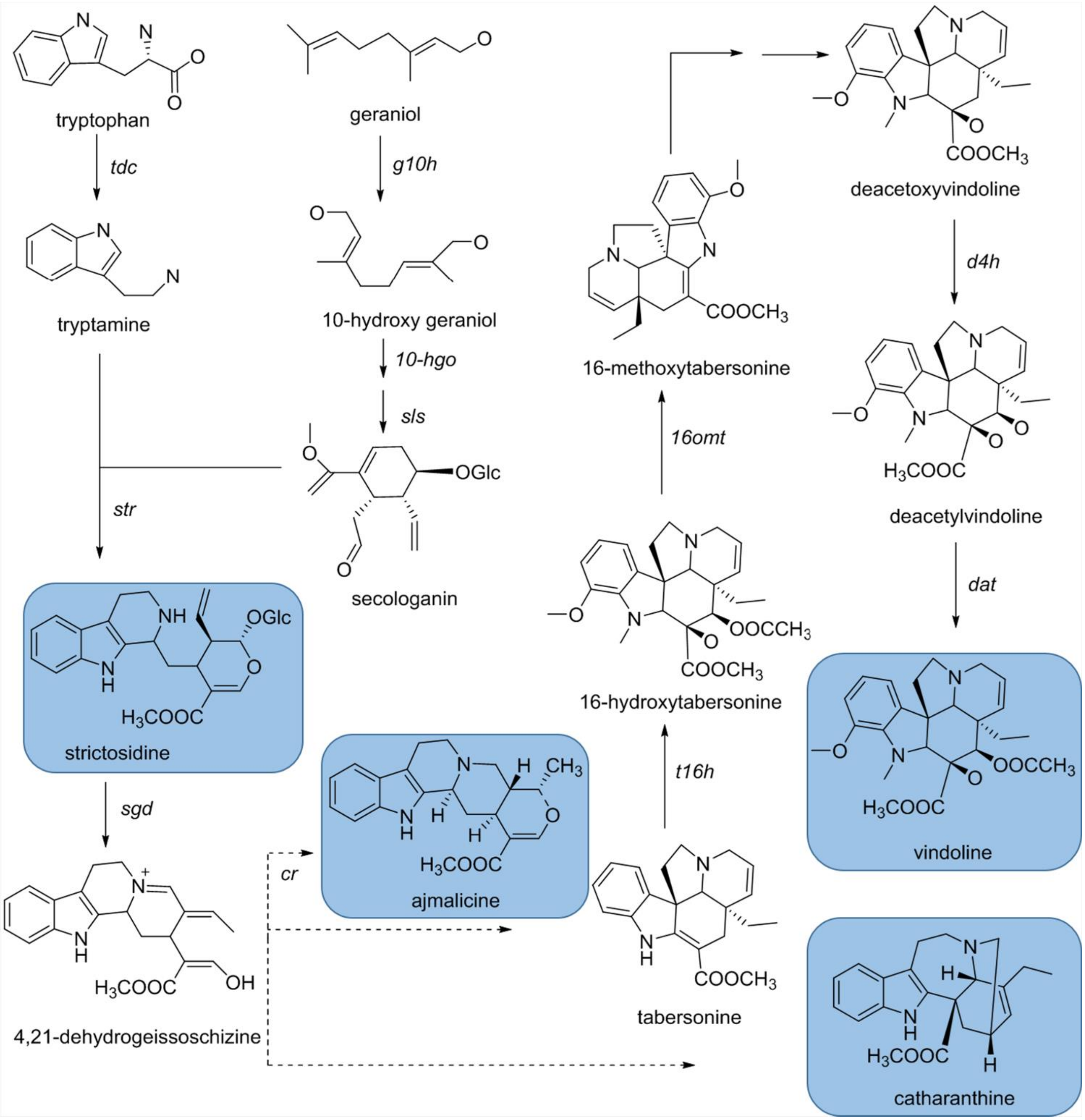

Supplementary Figure 4. The biosynthetic pathway of indole alkaloids in *C. roseus*. The induced alkaloids were marked by blue blocks. The alkaloid content is of alkaloids are shown in Supplemental Table 2. The abbreviations used are as follows: *tdc*, tryptophan decarboxylase; *g10h*, geraniol-10-hydroxylase; *10-hgo*, 10-hydroxygeraniol oxidoreductase; *sls*, secologanin synthase; *str*, strictosidine synthase; *sgd*, strictosidine β-D-glucosidase; *cr*, cathenamine reductase; *t16h*, tabersonine 16-hydroxylase; *16omt*, 16-hydroxytabersonine-16-O-methytransferase; *d4h*, deacetoxyvindoline 4-hydroxylase; *dat*, deacetylvindoline O-acetyltransferase.
